# Transcriptome Sequencing Reveals Differences between Primary and Secondary Hair Follicle-derived Dermal Papilla Cells of the Cashmere Goat (*Capra hircus*)

**DOI:** 10.1371/journal.pone.0076282

**Published:** 2013-09-19

**Authors:** Bing Zhu, Teng Xu, Jianlong Yuan, Xudong Guo, Dongjun Liu

**Affiliations:** The Key Laboratory of Mammalian Reproductive Biology and Biotechnology of the Ministry of Education, Inner Mongolia University, Hohhot, China; Casey Eye Institute, United States of America

## Abstract

The dermal papilla is thought to establish the character and control the size of hair follicles. Inner Mongolia Cashmere goats (*Capra hircus*) have a double coat comprising the primary and secondary hair follicles, which have dramatically different sizes and textures. The Cashmere goat is rapidly becoming a potent model for hair follicle morphogenesis research. In this study, we established two dermal papilla cell lines during the anagen phase of the hair growth cycle from the primary and secondary hair follicles and clarified the similarities and differences in their morphology and growth characteristics. High-throughput transcriptome sequencing was used to identify gene expression differences between the two dermal papilla cell lines. Many of the differentially expressed genes are involved in vascularization, ECM-receptor interaction and Wnt/β-catenin/Lef1 signaling pathways, which intimately associated with hair follicle morphogenesis. These findings provide valuable information for research on postnatal morphogenesis of hair follicles.

## Introduction

Dermal papilla cells (DPCs) are a population of mesenchymal cells at the base of the hair follicle (HF), and have become the focus of intense research interest because they are a key component that directly regulates HF development, growth and regeneration [1]. Communication between DPCs and the overlying epithelium is essential for initiation of hair cycling at the telogen phase, production of the hair shaft during the anagen phase, induction of follicle regression at the catagen phase and differentiation of HF lineages [2]. Key families of morphogenetic molecules mediate the above effects, including fibroblast growth factor (FGF), transforming growth factor-β (TGF-β), wnt pathway, sonic hedgehog (shh), neurotrophins, and homeobox gene families [3], [4], [5], [6].

Inner Mongolia Cashmere goat (*Capra hircus*) pledge skin contains two distinct types of HF, the primary hair follicle (PHF), which produces overhair or guard hair, and the secondary hair follicle (SHF), which produces underhair or cashmere [7], [8]. The follicle diameter and dermal papilla (DP) size of the PHF are much larger than those of the SHF. The post-natal HF of the Cashmere goat undergoes a circannual cycling of growth (anagen phase), regression (catagen phase) and rest (telogen phase) [9], [10], [11]. The growth cycle of PHF is similar with SHF, but at the end of telogen when moulting occurs and both the PHFs and SHFs shed their fibers, a sparse coat of mainly guard hair is maintained while the cashmere fibers detached almost completely [7], [10]. The long growth cycle and the obviously different size of the two types of follicle allows easy differentiation of the different hair cycle phases and HF types. These characteristics make the Cashmere goat an ideal model system for studies of HF morphology and development.

The DPs are thought to control the number of matrix cells and thus the character and size of the HF and its shaft [12], [13], [14]. Differentially expressed genes of DPCs among different HFs may be involved in morphological regulation of HFs. Rutberg identified four differentially expressed genes (*sfrp-2*, *mn1*, *atp1β1* and *fibulin-1d*) as specific biomarkers distinguishing human beard DPCs from scalp DPCs [15]. Two other dermally expressed genes, *sox18* and *sox2*, are involved in specifying HF types of mouse skin [2], [16]. However, relatively few DPC genes are known to be associated with the regulation of HF morphology. A full-scale investigation of differential gene expression of DPCs from two distinct, but closely located, types of HFs (the PHFs and SHFs of Cashmere goat) will identify genes potentially involved in HF morphogenesis.

In the present study, we obtained sufficient quantities of PHFs and SHFs separately from Cashmere goat skin during the anagen phase to isolate the dermal papillae from PHFs and SHFs. We then established the two cell lines and identified the differences between their transcriptome profilings using high-throughput sequencing. Differentially expressed genes between PHF-DPCs and SHF-DPCs will be very useful for further characterization of novel molecules associated with regulation of HF and hair shaft morphology.

## Materials and Methods

### Ethics statement

All studies adhered to procedures consistent with the International Guiding Principles for Biomedical Research Involving Animals issued by the Council for the International Organizations of Medical Sciences (CIOMS.) and were approved by the Institutional Animal Care and Use Committee at Inner Mongolia University. This study was also permitted by the owner of the YiWei White Cashmere Goat Farm.

### Isolation and culture of DP cells

This work was performed in September, during the anagen phase of the Cashmere goat hair growth cycle. Before surgery, ketamine (0.01 g/kg) was used to anesthetize six 2-year-old breeding female Inner Mongolia Cashmere goats, from YiWei White Cashmere Goat Farm in the Inner Mongolia Autonomous Region of China. Small excised parts of body side skins were depilated, washed and sterilized with 75% alcohol five times. Under a stereomicroscope, the PHFs and SHFs from the six goats' skins were isolated *in vitro* by microseparation. Both the PHFs and the SHFs were divided into six groups according to their source. After 30 min digestion in DMEM/F12 medium containing 0.2 mg/ml collagenase II (Gibco, Carlsbad, CA, USA) at room temperature, the DPs from PHFs or SHFs were separately microdissected by forceps and syringe needles. Subsequently, DPs from each group of PHFs or SHFs were added to 24-well culture plates (one DP per well) separately, and cultured in DMEM/F12 medium (plus 10% fetal calf serum) in 95% air/5% CO_2_ at 37°C. Cell cultures were observed and media were replaced every two days as the cells migrated from the papillae.

### Growth curves of PHF-DPCs and SHF-DPCs

Cells were seeded on 24-well culture plates at 1.0×10^4^ cells/ml. Cell numbers and cell density of each well were counted and recorded daily. The control comprised dermal fibroblast cells (DFCs), which were also obtained from Inner Mongolia Cashmere goat during the anagen phase. Cell numbers in four wells were counted at each time point, and the averages were used to plot the cell growth curve. Systat SigmaPlot 12.3 (http://www.systat.com/) plotted the curve. The mean population doubling times were estimated for the period of most rapid growth (between 3 and 19 days for DPCs and between 3 and 6 days for DFCs) and calculated as described by Oliver *et al*. Each experiment was repeated three times.

### Immunocytochemistry

PHF-DPCs or SHF-DPCs at the second passage grown on coverslips were washed with 0.01 M phosphate-buffered saline (PBS) and fixed in 4% paraformaldehyde for 15 min at 4°C. After rinsing three times with 0.01 M PBS, the fixed cells were treated with 0.2% Triton X-100 for 20 min at room temperature and rinsed again. The coverslips were then immersed in 3% hydrogen peroxide for 15 min at room temperature to block endogenous peroxidase activity. After washing in PBS, the coverslips were covered with 5% BSA in PBS for 20 min at room temperature to minimize nonspecific staining. Excess BSA was removed and the coverslips were covered with a solution containing a mouse monoclonal antibody [1A4] against alpha smooth muscle Actin (α-SMA) antibody (FITC) (Abcam, Cambridge, MA, USA), a rabbit polyclonal antibody against laminin (Wuhan Boster Biotech, Wuhan, China) or a rabbit polyclonal antibody against COL4A1 (Wuhan Boster Biotech) at 1∶100 dilution in PBS and incubated overnight at 4°C. An equal volume of PBS was added to the negative controls instead of primary antibodies. After washing in PBS three times, coverslips were covered with goat anti-rabbit IgG (Cy3) secondary antibody (Wuhan Boster Biotech) at a 1∶50 dilution and incubated for 50 min at room temperature (anti-α-SMA antibody [1A4] (FITC) omits this step), before being incubated for 10 min at room temperature with a 1 mg/L solution of 4′, 6′-diamidino-2-phenylindole (DAPI) (Wuhan Boster Biotech). Fluorescence images were obtained using Carl Zeiss AX10 inverted phase microscope with epifluorescence and a digital imaging system.

### Transcriptome profilings of PHF-DPCs and SHF-DPCs

About 1.0×10^7^ PHF-DPCs and 1.0×10^7^ SHF-DPCs at the second passage were obtained from the six groups of cultured PHF-DPCs and six groups of cultured SHF-DPCs, respectively. For each sample, cells were collected averagely from the different groups and pooled together. Total RNAs were isolated from each sample using a TRIzol Plus RNA Purification Kit according to the manufacturer's protocol (Invitrogen, Carlsbad, California, USA). Total RNA purity and concentration were determined using a 2100 Bioanalyzer Nanochip (Agilent Technologies, Palo Alto, CA, USA). The RNA-Seq libraries were constructed as previously described [17]. An Illumina/Solexa HiSeq2000 platform was used to sequence the RNA-Seq libraries. The raw reads were filtered to remove the adaptor sequences, low quality reads (>2% base smaller than Q20 per read) and reads containing undetermined bases (>2% 'N's per read was removed). The cleaned, high quality reads from PHF-DPCs and SHF-DPCs were aligned against the *Capra hircus* genome (NCBI PRJNA158393) assembly using TopHat [18]. Cufflinks was used to generate transcript annotation files and Cuffdiff [19], [20], [21] was used to measure the fragments per kilobase of transcript per million fragments mapped (FPKM) value for each protein-coding gene in the two types of DPCs. The differentially expressed genes between two samples were selected using the following criteria: i) if the FPKM value for a certain gene in both samples was greater than 1, the difference between them should be at least twofold. ii) If the FPKM value for a certain gene in one sample was less than 1, the FPKM value for this gene in the paired sample should be greater than 2. The goat genome assembly, genome annotation file and protein-encoding gene sequence can be obtained from the Goat Gene Database (http://goat.kiz.ac.cn/GGD/). The initial Illumia short reads generated by HiSeq2000 system in this study have been submitted to the NCBI Sequence Read Archive (SRA) under accession numbers SRX327891 (PHF-DPCs) and SRX327892 (SHF-DPCs).

### Quantitative real time PCR (qRT-PCR) analysis for validation of RNA-Seq data

Total RNA was extracted from the second passage PHF-DPCs and SHF-DPCs, respectively, using TRIzol Plus RNA Purification Kit (Invitrogen) following the manufacturer's protocols. The total RNA obtained was resuspended in nuclease-free water and the concentration was measured using a UV spectrophotometer (NanoDrop 2000, Thermo Scientific, Hudson, NH, USA). Total RNA were firstly treated with DNase I before reverse transcription by superscript III double-stranded cDNA synthesis kit (Invitrogen). Ten differentially expressed genes were selected randomly for validation of RNA-Seq data. QRT-PCRs were carried out on an ABI 7300 real-time PCR system (Applied Biosystems, Foster City, CA, USA) with SYBR Premix Ex Taq II kit (Takara, Kyoto, Japan). The primers used for qRT-PCRs analysis are listed in Table S3. Three biological replicates for each sample were used for this analysis. *Cnpy2* and *rnf10*, both of which had an equal FPKM value between PHF-DPCs and SHF-DPCs, were chose as internal reference genes to eliminate sample-to-sample variations. The relative gene expression levels were calculated using the 2^−ΔΔCt^ method [22]. The correlation coefficient (Pearson) of differential expression ratios between RNA-Seq and qRT-PCR was analyzed by using SPSS software 18.0 (http://www-01.ibm.com/software/analytics/spss/). The first group of qRT-PCR result (using *cnpy2* as internal reference gene) was selected for this analysis.

## Results

### Establishment and growth pattern of PHF-DPC and SHF-DPC lines

**Figure 1:**
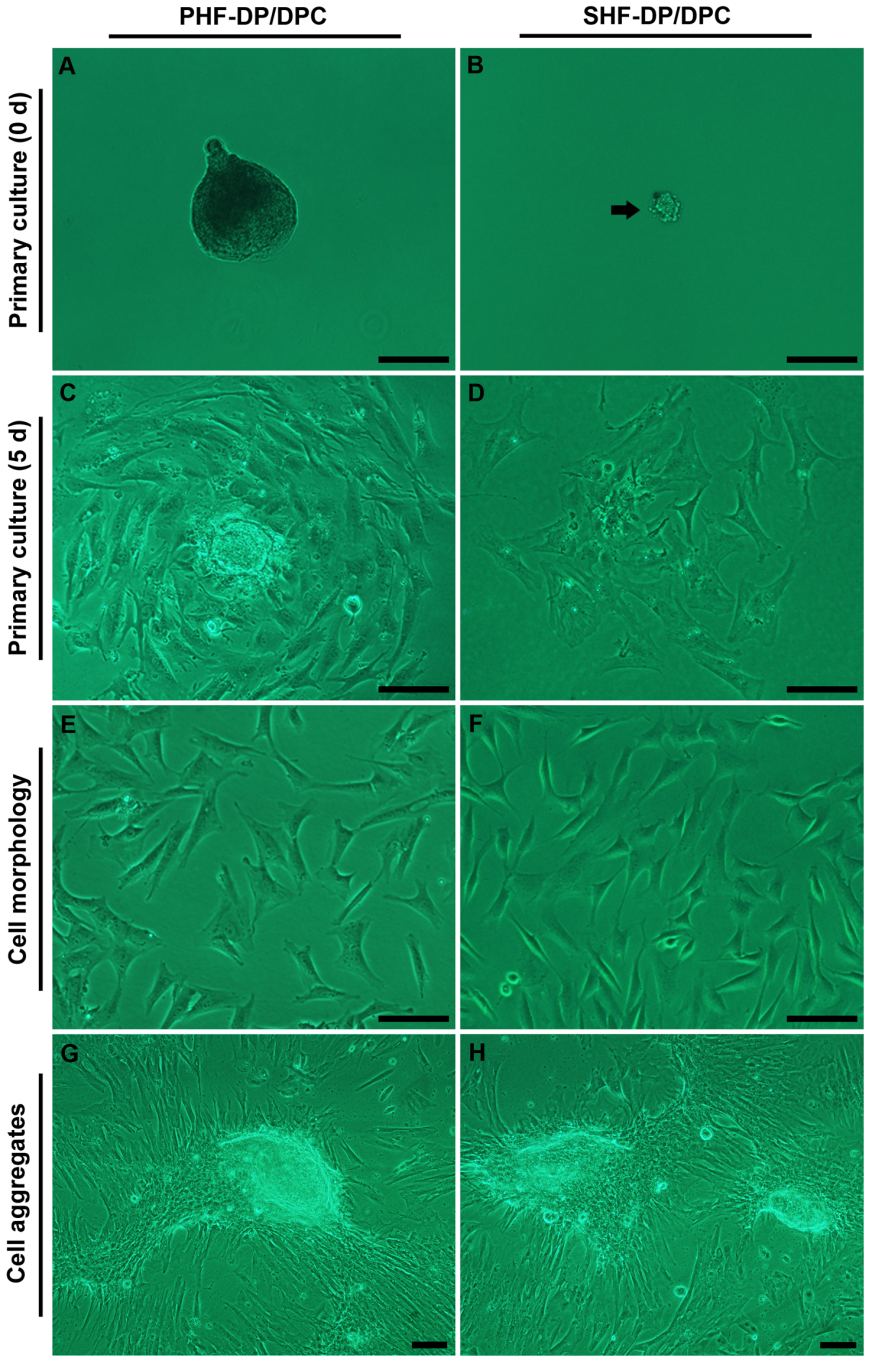
Culture of dermal papilla cells (DPCs) in the DMEM/F12 Medium plus 10% newborn calf serum. Primary culture of the primary hair follicle-dermal papilla cells (PHF-DPCs) (A, C), and secondary hair follicle-dermal papilla cells (SHF-DPCs) (B, D). The DPCs exhibited a triangular or polygon shape (Figure 1C–F) at primary and subsequent passage. At the second passage both the PHF-DPCs (E) and SHF-DPCs (F) formed cell aggregates during further culture for approximately 20 days. Black arrow in (B) shows the tiny DP isolated from SHF. Scale bars  = 100 µm.

We isolated and established two DPC lines, a PHF-DPC line and a SHF-DPC line, by microdissection and collagenase digestion of PHFs and SHFs. Both cell lines were passaged over 20 times. Figure S1 showed the process of DP microdissection. Finally, DPs from PHFs (Figure 1A) and SHFs (Figure 1B) were transferred into media for primary culture.

Cells migrated from the papillae after 5 d in culture (Figure 1C, D). The DPCs exhibited a triangular or polygon shape (Figure 1C–F) and an aggregative growth behavior at primary and subsequent passages. Cell aggregates were formed with further culture for about 20 days (Figure 1G, H). The DPCs did not lose their aggregative ability even up to the 20th passage of both PHF-DPCs and SHF-DPCs, which is much longer than that of rat vibrissa DPCs [23]. This indicated that DPCs from Cashmere goats might possess a more enduring ability for HF induction [23], [24].

**Figure 2:**
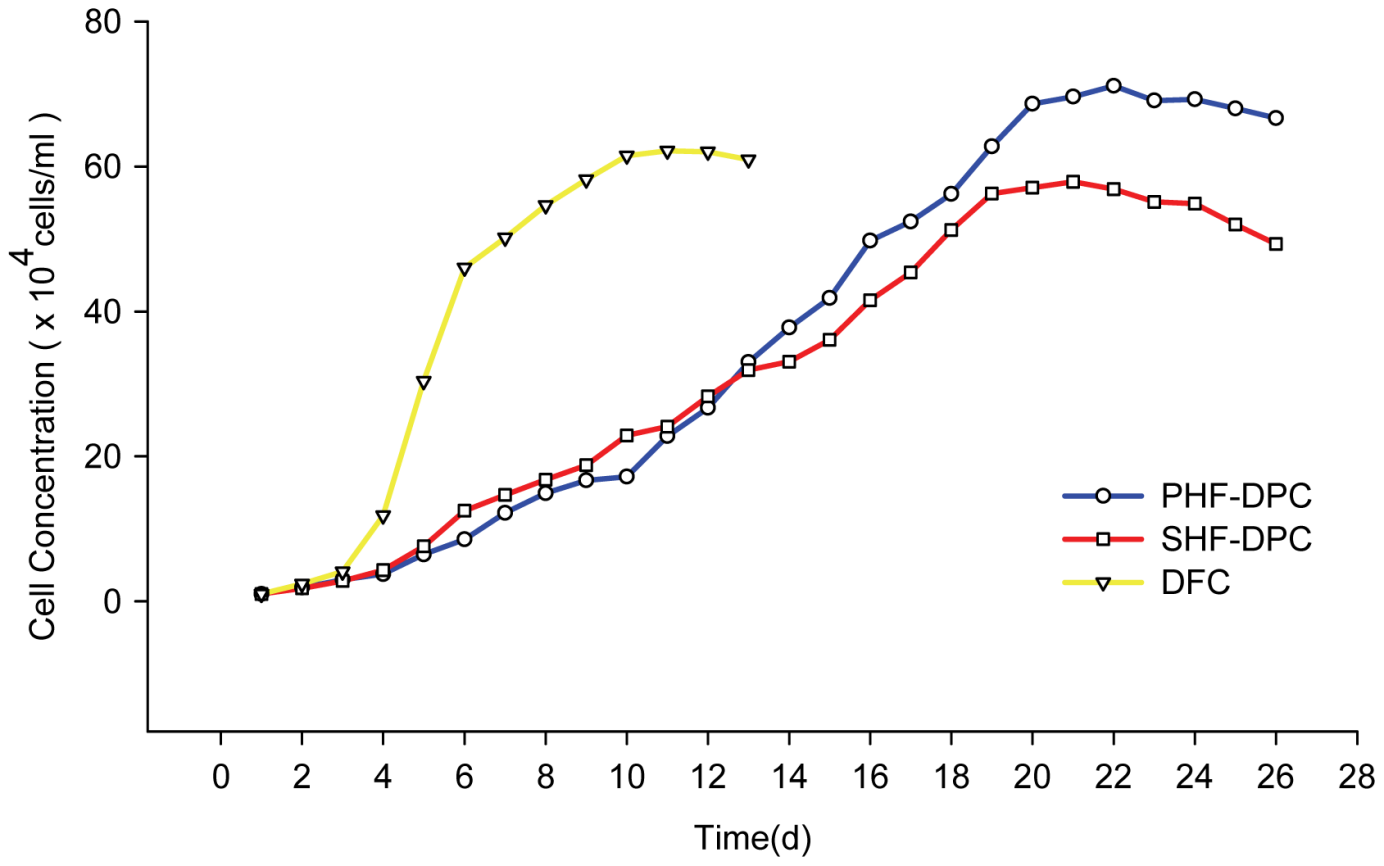
Growth curves of primary hair follicle-dermal papilla cells (PHF-DPCs), secondary hair follicle-dermal papilla cells (SHF-DPCs) and dermal fibroblast cells (DFCs). The inoculum was 1.0×10^4^ cells/ml. Cell numbers were counted daily and recorded. The population doubling time was approximately 3.6days for PHF-DPC, 3.7days for SHF-DPC and 0.86days for DFC.

We analyzed the growth patterns of PHF-DPC and SHF-DPC, using DFC as a control (Figure 2). PHF-DPCs and SHF-DPCs had similar growth rates, but were significantly different from the DFCs. Both types of DPCs took about 21 days to reach their maximum cell density in culture, whereas it took about 10 days for the DFCs. The maximum cell density reached by PHF-DPCs was about 1.23-fold than SHF-DPCs. Mean population doubling time was 3.60 days for PHF-DPCs, 3.70 days for SHF-DPCs and 0.86 days for the DFCs. PHF-DPCs (Figure 1G) seemed to form aggregates a little faster and were larger than those of SHF-DPCs (Figure 1H).

### Identification of the DPCs using specific antibodies

**Figure 3:**
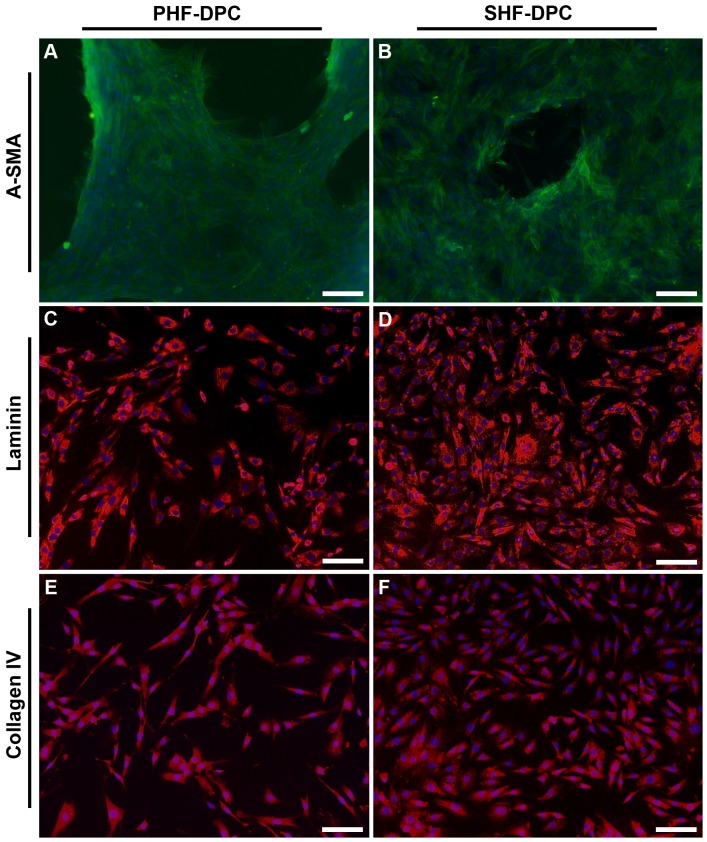
Immunocytochemical analysis of cultured dermal papilla cells (DPCs). Immunocytochemistry of DPCs using anti-α-SMA antibody (green) was performed (A&B) when cells started to exhibit an aggregative growth behavior, while the other two antibodies were performed on monolayer cultured DPCs. Both primary hair follicle-dermal papilla cells (PHF-DPCs) and secondary hair follicle-dermal papilla cells (SHF-DPCs) were positive for α-SMA (green, A & B), Laminin (red, C & D), and Collagen IV (red, E & F). Nuclei in A–F were marked by DAPI staining (blue). Scale bars  = 100 µm.

We examined the expression patterns of three genes in the two types of cultured DPCs at the sixth passage using immunocytochemistry. As shown in Figure 3A and B, the presence of blank spaces suggested that the growth of both PHF-DPCs and SHF-DPCs exhibited an aggregative behavior. Subsequent examination showed that the anti-α-SMA antibody stained both PHF-DPCs (A, green) and SHF-DPCs (B, green) positively, suggesting that the two types of DPCs strongly expressed α-SMA, a marker of cultured DPCs [25], [26]. Laminin (Figure 3C and D, red) and collagen IV (Figure 3E and F, red), which are the components of the extracellular matrix (ECM), were also expressed in both types of DPC.

### Transcriptome profilings of PHF-DPCs and SHF-DPCs

To quantify the gene expression patterns of PHF-DPCs and SHF-DPCs, we constructed two RNA-Seq libraries for the two independently cultured cell lines and then subjected them to deep sequencing using Illumina/Solexa technology. In total, we obtained 51,818,210 and 45,637,260 reads from PHF-DPCs and SHF-DPCs, respectively. Among them, 46,365,560 and 40,989,751 short reads could be mapped to the goat reference genome (Scaffold), and 13,248 and 13,139 transcripts from 22,175 well-annotated goat protein-encoding genes were detected as expressed using FPKM value (FKPM>0) in PHF-DPCs and SHF-DPCs (Table S1), respectively. Among these genes, Heat shock protein β-1 (GENE_ID: GOAT_ENSBTAP00000015883) was the most abundant transcript, with an FPKM score >400,000 in both types of DPC. Other high expression level transcripts in the two samples included various ribosome proteins, ubiquitin, which indicated a high level of protein synthesis and degradation in the DPCs. We also noted that the Agouti-signaling protein (GENE_ID: GOAT_ENSP00000364092), a paracrine signaling molecule that causes the synthesis of pheomelanin in the melanocytes of the HF [27], was highly expressed (FPKM>8000), suggesting that DPC is the major signal transmitter for directing pheomelanin generation.

**Figure 4:**
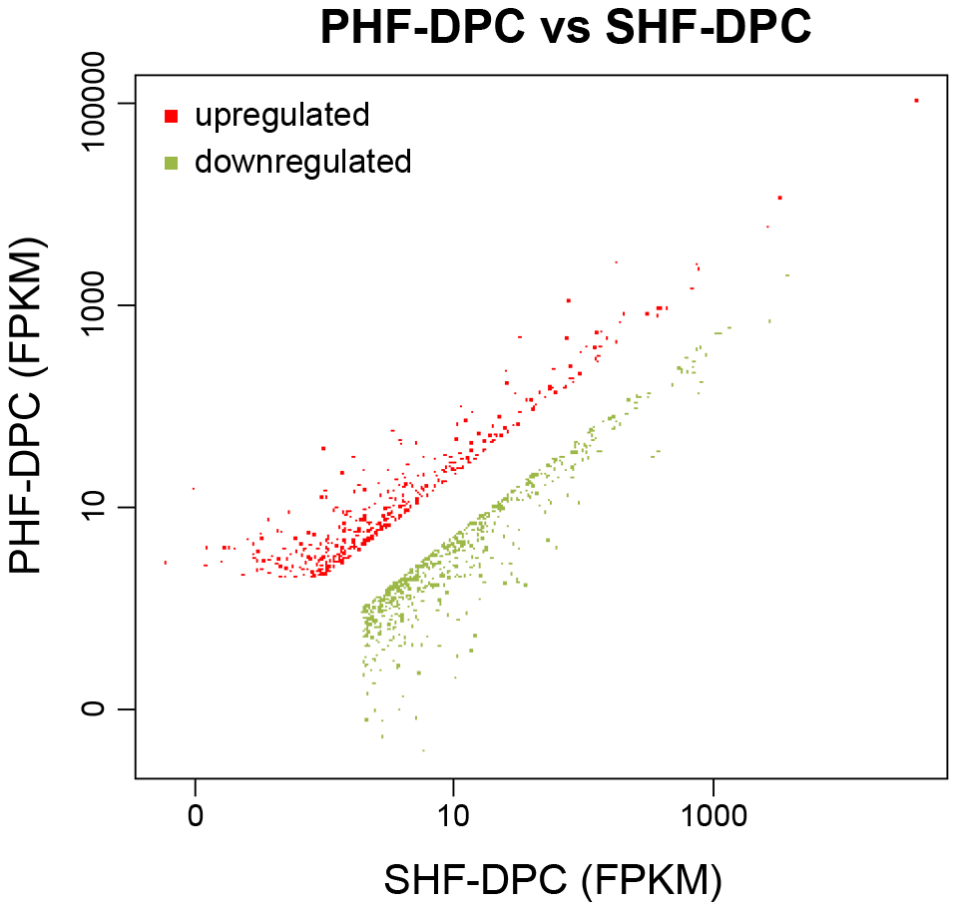
Schematic representation of the differentially expressed genes between primary hair follicle-dermal papilla cells (PHF-DPCs) and secondary hair follicle-dermal papilla cells (SHF-DPCs). Of the 1044 differentially expressed genes, 620 were upregulated (top-left, red) and 424 were downregulated (bottom-right, green) in PHF-DPCs compared with SHF-DPCs.

To identify gene expression differences between PHF-DPCs and SHF-DPCs, differential gene expression profiling was conducted. We identified 1044 genes that were expressed at least two-fold differently between the two types of DPC (Table S2). 620 genes from PHF-DPC were upregulated compared with SHF-DPC and 424 were downregulated (Figure 4).

### Validation of RNA-Seq data

**Figure 5:**
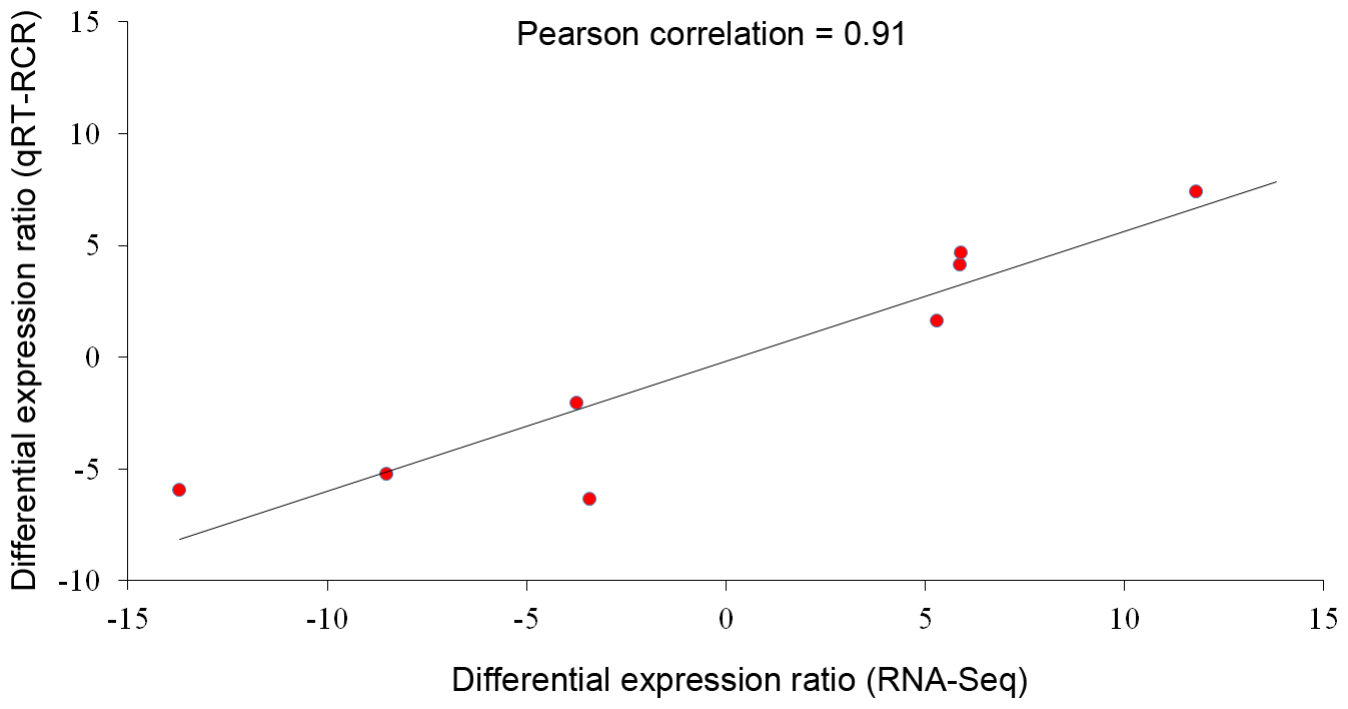
Pearson correlation coefficient^*^ of differential expression ratios between RNA-Seq and qRT-PCR. *Correlation is significant at the 0.01 level.

To validate results from transcriptomic analysis, ten differentially expressed genes were selected randomly and subjected to the qRT-PCR analysis. Among the genes tested, the expression of genes encoding IRK8 and EYA2 were too low to detected in the SHF-DPCs (FPKM = 0) by using RNA-Seq. However, they could be detected by qRT-PCR but had much lower expression levels than PHF-DPCs. Table 1 shows that the expression profiling of these differentially expressed genes by using qRT-PCR had the similar trends with RNA-Seq samples, both in two groups with individual internal reference gene. As the FPKM ratio of IRK8 and EYA2 between PHF-DPCs and SHF-DPCs were infinite (Table 1), this two genes was excluded for the Pearson correlation coefficient analysis. The Pearson correlation coefficient of differential expression ratios between RNA-Seq and qRT-PCR was 0.91 (Figure 5), indicating the gene expression differences observed in transcript abundance between PHF-DPCs and SHF-DPCs were highly credible.

**Table 1:** Validation of ten differentially expressed genes with biological replicates using qRT-PCR.

Method	Fold difference of gene abundance between PHF-DPCs and SHF-DPCs									
	MK	ILEU	TIG1	CAH11	HTRA3	COT2	ANXA8	CO3	IRK8	EYA2
RNA-Seq^a^	5.88	−3.75	−3.20	5.89	−14.1	5.30	11.80	−8.50	INF (9.6∶0)	INF (4.94∶0)
qRT-PCR/*cnpy2* b	4.42	−2.05	−6.87	4.68	−5.76	1.54	7.42	−5.24	27.05	15.95
qRT-PCR/*rnf10* c	5.57	−1.61	−5.14	6.32	−4.31	2.10	6.10	−4.46	36.57	21.56

a Fold difference for each gene was calculated by FPKM value of PHF-DPCs and SHF-DPCs.

b Using *cnpy2* as the internal reference gene to calculate relative gene expression level.

c Using *rnf10* as the internal reference gene to calculate relative gene expression level.

## Discussion

### Establishment of two DPC lines

DPCs have been cultured from many species [13], [15], [28], [29], [30], including rat whiskers, mouse vibrissa and pelage, sheep skin, and human beards and scalps. To the best of our knowledge, this is the first study to establish DP cell lines from Cashmere goats. Microdissection combined with collagenase II digestion is a potent and efficient method to isolate DPs from HFs. Interestingly, dispase appeared not to be useful for separating HFs from Cashmere goats, although it had great efficiency in handling mouse skin [31].

### Differentially expressed genes between PHF-DPC and SHF-DPC involved in vascularization and probable hair follicle morphogenesis

Inner Mongolia Cashmere goat pelage skin contains two kinds of HF. The most obviously morphological difference between PHFs and SHFs is the follicle size. Interactions between dermal and epidermal cells have been reported to play essential roles in HF morphogenesis and development [1], [2]. The DP is embedded at the bottom of the follicle, where it is surrounded by the hair matrix and differs in size among various follicular types. This suggested that the DP is the key component that directly regulates HF size [12]. In this study, we identified the genes that were differentially expressed between PHF-DPCs and SHF-DPCs at the transcript level, which will provide useful information for research on hair follicle morphogenesis.

Enhanced follicle vascularization can promote hair growth and increase HF size and hair diameter [32], [33]. A great number of differentially expressed genes involving in angiogenesis or antiangiogenesis between PHF-DPCs and SHF-DPCs are probably participated in the follicle microvascular formation process and subsequent hair follicle morphogenesis. Among these genes, 27 genes were upregulated in the PHF-DPCs, within 19 have promoting angiogenesis properties and 8 have suppressing properties (Table 2), while 23 genes were upregulated in the SHF-DPCs, within 17 have promoting angiogenesis effects and 8 have suppressing effects (Table 3). Some other differentially expressed genes, including those encoding ETS-related transcription factor Elf-1, Alpha-actinin-2, Integrin alpha/beta-3, Tenascin, and Mimecan, their functions also seem important in regulating or mediating the angiogenesis process [34], [35], [36], [37], [38], [39], [40]. Whereas, the role of Insulin-like growth factor-binding protein 3 in this process is still controversial [41], [42], [43], [44].

**Table 2:** Upregulated genes in PHF-DPCs that involved in angiogenesis.

GENE_ID	FPKM value		Fold difference	Gene Description	Function (Promoting or suppressing angiogenesis)
	PHF-DPC	SHF-DPC	P/S		
GOAT_ENSBTAP00000012804	2.69129	0	INF	Pigment epithelium-derived factor	Suppressing [83]
GOAT_ENSP00000385521	13.8691	0.376696	36.82	Fibulin-1 (Fragment)	Suppressing [84]
goat_GLEAN_10008671	3.48077	0.301979	11.53	Fibulin-1	Suppressing [84]
GOAT_ENSP00000222139	4.88972	0.427914	11.43	Erythropoietin receptor	Promoting [58], [59]
GOAT_ENSBTAP00000002983	388.34	35.0126	11.09	Pleiotrophin	Promoting [85], [86]
goat_GLEAN_10011961	32.7034	3.85736	8.48	Dysferlin	Promoting [87]
GOAT_ENSBTAP00000000993	7.94762	1.14915	6.92	Aquaporin-1	Promoting [88], [89]
GOAT_ENSBTAP00000010178	77.5807	12.8088	6.06	Midkine	Promoting [90]
GOAT_ENSP00000377721	57.2941	10.9036	5.25	COUP transcription factor 2	Promoting [91]
GOAT_ENSP00000002829	84.0437	19.2575	4.36	Semaphorin-3F	Suppressing [92]
GOAT_ENSP00000264634	14.6723	3.8305	3.83	Protein Wnt-5a	Promoting [93]
GOAT_ENSP00000365012	6.61417	1.74338	3.79	Tyrosine-protein kinase HCK	Promoting [53]
GOAT_ENSP00000422464	16.8709	4.86153	3.47	Platelet-derived growth factor C	Promoting [94]
GOAT_ENSBTAP00000013284	724.376	210.177	3.45	Early growth response protein 1	Suppressing [95]
GOAT_ENSP00000362299	83.2965	24.3401	3.42	Endoglin	Promoting [96]
GOAT_ENSBTAP00000009523	21.0185	6.82305	3.08	Tumor necrosis factor-inducible gene 6 protein	Promoting [97]
GOAT_ENSBTAP00000026901	2.16054	0.729603	2.96	Meteorin	Promoting [98], [99]
GOAT_ENSP00000248673	175.897	60.933	2.89	Tristetraprolin	Suppressing [100]
goat_GLEAN_10003882	11.7056	4.12153	2.84	Glutaredoxin-1	Promoting [101]
GOAT_ENSP00000244766	4.24482	1.58842	2.67	Neuritin	Promoting [102], [103]
GOAT_ENSBTAP00000006923	581.708	225.435	2.58	Osteopontin	Promoting [104], [105]
GOAT_ENSBTAP00000020424	31.2585	12.2317	2.56	Secreted frizzled-related protein 4	Suppressing [106]
GOAT_ENSP00000358045	9.29708	4.03879	2.30	Extracellular matrix protein 1	Promoting [107]
GOAT_ENSP00000358817	14.5448	6.76541	2.15	Macrophage colony-stimulating factor 1	Promoting [108]
GOAT_ENSBTAP00000050029	113.73	53.1482	2.14	Hypoxia-inducible factor 1-alpha	Promoting [109]
GOAT_ENSP00000370542	60.3296	29.7654	2.03	Syndecan-1	Promoting [110]
GOAT_ENSP00000235332	8.64092	4.29094	2.01	Migration and invasion-inhibitory protein	Suppressing [111]

**Table 3:** Upregulated genes in SHF-DPCs that involved in angiogenesis.

GENE_ID	FPKM Value		Fold Difference	Gene Description	Function in angiogenesis
	PHF-DPC	SHF-DPC	S/P		
GOAT_ENSBTAP00000006610	0.204515	3.85086	18.83	Semaphorin-5A	Promoting [112]
GOAT_ENSP00000297904	0.542289	8.75929	16.15	Vascular endothelial growth factor D	Promoting [46]
GOAT_ENSBTAP00000052221	183.105	2575.06	14.06	Serum amyloid A protein	Promoting [113], [114]
GOAT_ENSBTAP00000030311	78.0688	1083.56	13.88	Serum amyloid A protein	Promoting [113], [114]
GOAT_ENSP00000381891	0.974225	12.3189	12.64	Transcriptional regulator ERG	Promoting [115]
GOAT_ENSBTAP00000007349	75.6252	460.304	6.90	Insulin-like growth factor-binding protein 2	Promoting [116], [117]
GOAT_ENSP00000167106	0.669865	4.27558	6.38	Vasohibin-1	Suppressing [118], [119]
GOAT_ENSBTAP00000002997	1.41533	8.82916	6.24	Myelin basic protein	Promoting [120]
GOAT_ENSP00000369071	1.33357	6.28977	4.72	Periostin	Promoting [121], [122]
GOAT_ENSP00000287641	2.49128	10.7254	4.31	Somatostatin	Suppressing [123], [124]
GOAT_ENSP00000355751	30.625	129.848	4.24	Thrombospondin-2	Suppressing [60], [61]
GOAT_ENSP00000356281	3.85378	16.1375	4.19	Troponin I, slow skeletal muscle	Suppressing [125]
GOAT_ENSBTAP00000022540	0.727229	2.80047	3.85	Type-1 angiotensin II receptor	Promoting [126]
GOAT_ENSP00000406949	22.8084	77.4927	3.40	Elastin	Promoting [127]
GOAT_ENSBTAP00000026471	2.19641	6.95152	3.16	Neuropilin-1	Promoting [48], [49]
GOAT_ENSP00000272928	11.3771	33.8995	2.98	C-X-C chemokine receptor type 7	Promoting [128], [129]
GOAT_ENSBTAP00000007931	3.49304	10.2859	2.94	WNT1-inducible-signaling pathway protein 2	Promoting [130]
GOAT_ENSP00000365280	21.3007	51.3928	2.41	DNA-binding protein inhibitor ID-1	Promoting [131]
GOAT_ENSBTAP00000008357	408.494	921.63	2.26	Connective tissue growth factor	Suppressing [132], [133]
GOAT_ENSBTAP00000021062	12.022	25.2465	2.10	Tissue factor pathway inhibitor 2	Suppressing [134]
GOAT_ENSP00000254958	2.39325	4.94465	2.07	Protein jagged-1	Promoting [135], [136]
GOAT_ENSP00000385465	26.7012	55.3307	2.07	DNA-binding protein inhibitor ID-2	Promoting [137]
GOAT_ENSP00000346839	381.57	772.144	2.02	Fibronectin	Promoting [138], [139]

Among four VEGF family members, VEGF-A, -C and -D are believed to promote angiogenesis (or lymphangiogenesis) and vascular permeability, with VEGF-D being the most potent, causing a remarkable enlargement of microvessels maintaining proper vasculature around the hair follicle during the anagen of hair growth cycle [45], [46], [47]. The expression of VEGF-D was upregulated by about 16-fold in SHF-DPC, although its transcript level in both PHF-DPCs and SHF-DPCs was much lower than that of VEGF-A and -C, both of which showed no significant differences between PHF-DPCs and SHF-DPCs. Higher expression of VEGF-D in SHF-DPCs may be related to improved follicle vascularization and subsequent folliculomorphogenesis.

Other upregulated genes in SHF-DPCs include Neuropilin-1, which is a novel cell-surface receptor of VEGF that mediates VEGF-dependent angiogenesis in mice and zebrafish [48], [49]. It is upregulated via the VEGFRII-dependent pathway [50]. Selective inhibition of Neuropilin-1 suppressed neovascular formation substantially in a murine model [50].

Hemopoietic cell kinase (HCK) is a member of the Src family, which is expressed in neutrophils [51], [52]. We found that the HCK was also expressed in both DPCs. It has been reported that *hck^−/−^fgr^−/−^* mice were unable to develop an angiogenic response or release VEGF-A upon stimulation with CXCL1/MIP-2 [53]. Upregulated HCK in PHF-DPCs may have a positive function in the induction of follicle vascularization.

EPO (erythropoietin) is a glycoprotein hormone that controls the proliferation, differentiation and survival of erythroid progenitor cells [54]. HFs are believed to be extrarenal sites of EPO production and extrahematopoietic sites of EPO receptor (EPO-R) expression, where EPO/EPO-R signaling occurs [55]. The EPO protein is exclusively produced by the outer root sheath during the anagen phase in HFs [55]. Organ-cultured human scalp HFs showed an upregulated expression pattern of hemoglobin alpha-1 upon stimulation with EPO [55]. DPs are one of the targets of EPO [56], and its EPO-R respond to EPO with enhanced cell proliferation [57]. Correlations between EPO/EPO-R levels and angiogenesis were reported in clinical studies [58], [59], but not completely clarified. We suggest that the upregulated EPO-R in PHF-DPCs may reflect increased EPO stimulation compared with SHF-DPCs, resulting in subsequent promotion of angiogenesis around the PHFs.

The thrombospondins (TSPs) are a family of adhesive molecules that influence the attachment, migration and growth of a variety of cell types [60]. Both TSP-1 and TSP-2, encoded by *thbs-1* and *-2*, respectively, are matricellular glycoproteins [61] and have been reported to have potent antiangiogenic properties. Other members, such as TSP-3, TSP-4 or TSP-5/COMP, do not show this property [60], [62]. *Thbs-1* had a similar expression pattern in PHF-DPCs and SHF-DPCs, whereas *thbs-2* was highly upregulated in SHF-DPCs. The antiangiogenic activity of both TSP-1 and -2 are mediated by the binding of thrombospondin type 1 repeats (TSR) to CD36 [61]. However, the antiangiogenic activity of TSP-2, unlike TSP-1, lacks the capacity to activate TGF-β [61], [63], which has a crucial inductive ability in vascular formation when combined with its signaling mediators [64], [65], [66]. This suggests that TSP-2 has a special mode in antiangiogenesis that is different from the role of TSP-1, to avoid neutralization by the angiogenic activity induced by TGF-β. The higher expression of *thbs-2* may inhibit follicle vascular formation around SHF-DPCs *in vivo*. However, the biological roles of TSP-1 and -2 remain elusive [67].

**Figure 6:**
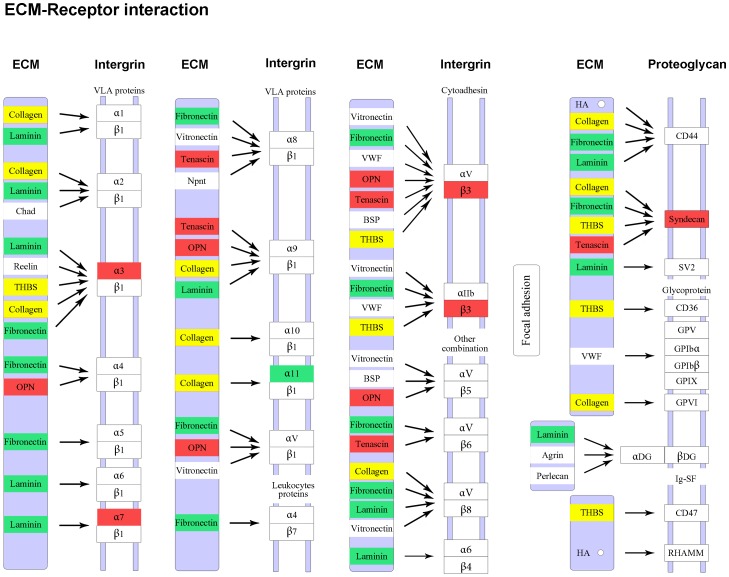
Differentially expressed genes between primary hair follicle-dermal papilla cells (PHF-DPCs) and secondary hair follicle-dermal papilla cells (SHF-DPCs) involved in extracellular matrix (ECM)-receptor interaction pathway. The red color labels genes upregulated in the PHF-DPCs compared with the SHF-DPCs. The green color labels genes downregulated in the PHF-DPCs. The yellow color labels genes that some were upregulated and others were downregulated in the PHF-DPCs.

Angiogenesis not only depends on these specific molecules, but is also influenced by receptors for ECM proteins [66], e.g., integrins and proteoglycans. We found that a number of these receptors, which are expressed on the surface of DPCs and could regulate critical adhesive interactions with ECM proteins, were also differentially expressed, e.g., Integrin a3, a11, β3, and Syndecan (Figure 6 and Table 4).

**Table 4:** Expression level of differentially expressed genes between PHF-DPCs and SHF-DPCs involved in ECM-receptor interaction pathway.

Gene_id	FPKM value		Fold difference	Protein name	Discription
	PHF-DPCs	SHF-DPCs	P/S		
GOAT_ENSBTAP00000000063	2.90024	0.82487	3.52	TSP3	Thrombospondin-3
GOAT_ENSBTAP00000000752	494.26	158.242	3.12	TENA	Tenascin
GOAT_ENSBTAP00000001200	3.63005	0.715031	5.08	CORA1	Collagen alpha-1(XXVII) chain
GOAT_ENSBTAP00000006923	581.708	225.435	2.58	OSTP	Osteopontin
GOAT_ENSBTAP00000013173	3.96248	1.7004	2.33	ITB3	Integrin beta-3
GOAT_ENSBTAP00000017783	0.966253	2.63545	−2.73	COEA1	Collagen alpha-1(XIV) chain
GOAT_ENSBTAP00000039682	2.25669	9.78232	−4.33	FNDC1	Fibronectin type III domain-containing protein 1
GOAT_ENSP00000007722	21.2234	10.0458	2.11	ITA3	Integrin alpha-3
GOAT_ENSP00000343009	2.58019	0.340288	7.58	ITA7	Integrin alpha-7
GOAT_ENSP00000346839	381.57	772.144	−2.02	FINC	Fibronectin
GOAT_ENSP00000348384	0.558045	2.32622	−4.17	LAMB3	Laminin subunit beta-3
GOAT_ENSP00000355751	30.625	129.848	−4.24	TSP2	Thrombospondin-2
GOAT_ENSP00000370542	60.3296	29.7654	2.03	SDC1	Syndecan-1

### PHF-DPCs and SHF-DPCs differ in the expression of ECM genes

The ECM is composed of a network of fibrous structural proteins (e.g., collagen, laminin, elastin, and fibronectin) that form macromolecular structures as their functional embodiments, and matricellular proteins (e.g., TSPs and tenascins) that not directly contribute to the formation or function of structural complexes, but modulate cell-matrix interactions and cell functions [68]. ECM-receptor interaction is essential for morphogenesis of tissues and organs, playing roles in maintaining their structural and functional homeostasis, and in the control of gene expression [69], [70], [71], [72]. Specific cell surface molecules, such as Integrin, Proteoglycans, CD36, and CD44, mediate these functions by ECM-cell adhesion, cell migration, differentiation, proliferation, and apoptosis. Our previous studies showed that high expression of ECM and cell surface proteins was essential for the rapid growth of Cashmere goat HFs during the anagen phase [17]. Campbell *et al*. particularly showed the role of ECM in the angiogenic process [73], in which the major ECM proteins include collagen, laminin and fibronectin mediated angiogenesis through arginine-glycine-aspartic acid (RGD) motifs which bind to integrins to mediate signaling.

The volume of the DP depends on the number of cells it contains and the amount of ECM per cell [74], which regulates cell proliferation, migration, adhesion, and aggregation [75], [76]. In this study, we found that the ability of cultured PHF-DPCs to form cell aggregates seemed higher than in cultured SHF-DPCs (Figure 1-G & -H), which is consistent with the size of DPs in PHFs and SHFs *in vivo*. Many of the ECM genes, including *collagen*, *laminin*, *thbs*, and *fibronectin* were differentially expressed between PHF-DPCs and SHF-DPCs (Figure 6), indicating that the expression of these genes probably resulted in a higher amount of ECM in PHF-DPCs, which contributes to the larger size of the DP. However, these observation or supposition requires further research. Table 4 shows the differentially expressed genes involving in the ECM-receptor interaction pathway.

Androgens, as one of the hormones involved in ECM-receptor interaction, have the most dramatic effects on the size of HFs, acting through androgen receptors in the DP [12]. However, its effect mainly appears in androgen-dependent areas, such as the beard [12]. The expression of the androgen receptor in both types of DPC was low and showed no significant difference. It seems that the androgens are not critical to the morphological difference between PHFs and SHFs.

The microvascular system and ECM together constitute the microenvironment around the HF, which may regulate the structure, metabolism and signaling of DPs. Differences in the microenvironment between PHF-DPs and SHF-DPs may provide information on controlling and regulating of the follicle morphogenesis.

### Differentially expressed genes involving in Wnt/β-catenin/Lef1 signaling pathways associated with HF morphogenesis

Wnt signaling has been demonstrated to be absolutely necessary for the initiation and morphogenesis of all types of HF [77]. The FPKM value of Wnt repressor Dickkopf-1 was reduced to zero both in PHF-DPCs and SHF-DPCs, while Wnt5α (GOAT_ENSP00000264634) and Wnt5β (GENE_ID: GOAT_ENSBTAP00000001766) were upregulated in PHF-DPCs. Recent studies demonstrated that Wnt5α attenuates the β-catenin signaling pathway and represses the expression of Lef1 (GENE_ID: GOAT_ENSBTAP00000008991) in DPCs [78]. In fact, the expression of Lef1 (induced by the BMP4 antagonist Noggin [79]), which has been disclosed to be essential for the secondary HF development [79], [80], was indeed downregulated in the PHF-DPCs while upregulated in the SHF-DPCs. Stabilized (by Wnt signaling [81]) nuclear β-catenin can form complexes with various Tcf/Lef1 DNA-binding proteins to activate downstream target gene sets [82]. Modifications of these signalings in DPCs may lead to the different gene expression regulation, and subsequently, affect the development and morphogenesis of PHFs and SHFs.

## Conclusions

The pelage skin of the Inner Mongolia Cashmere goat has two types of HF, which differ in their follicle diameter and DP size. DP cell lines from the two types of HF (PHF and SHF) during the anagen phase were successfully established. Gene expression patterns of the two DP cell lines were analyzed, and the differences suggested that those genes involving in vascularization, ECM-receptor interaction and Wnt/β-catenin/Lef1 signaling pathways might together regulate DP size and HF morphology. The key differentially expressed genes may be considered as potential candidate genes for further study on the regulation of hair follicle development and morphogenesis.

## Supporting Information

Figure S1 **The process of DP microdissection.** A) The originally isolated PHFs and SHFs. The SHFs grow in bunches (difficult to separate from each other) while the PHFs do not. The PHFs are spaced apart from each other by the dermal fibers *in vivo*. In this study, only the mature hair follicles which were easily to distinguish from each other were sampled. B), C), and D) showed the microdissection process of DP (Black arrow in C) from PHF. Scale bar  = 250 µm.
(TIF)

Table S1 Transcripts level abundance in PHF-DPCs and SHF-DPCs.
(XLS)

Table S2 Differentially expressed genes between PHF-DPCs and SHF-DPCs.
(XLS)

Table S3 Primers used for the qRT-PCR analyses.
(XLS)
