# Supplementary figures and images for: Transcriptome Sequencing Reveals Differences between Primary and Secondary Hair Follicle-derived Dermal Papilla Cells of the Cashmere Goat (Capra hircus)
Source: PLoS One. 2013 Sep 19;8(9):e76282. doi: 10.1371/journal.pone.0076282 (PMC3777969; doi:10.1371/journal.pone.0076282)

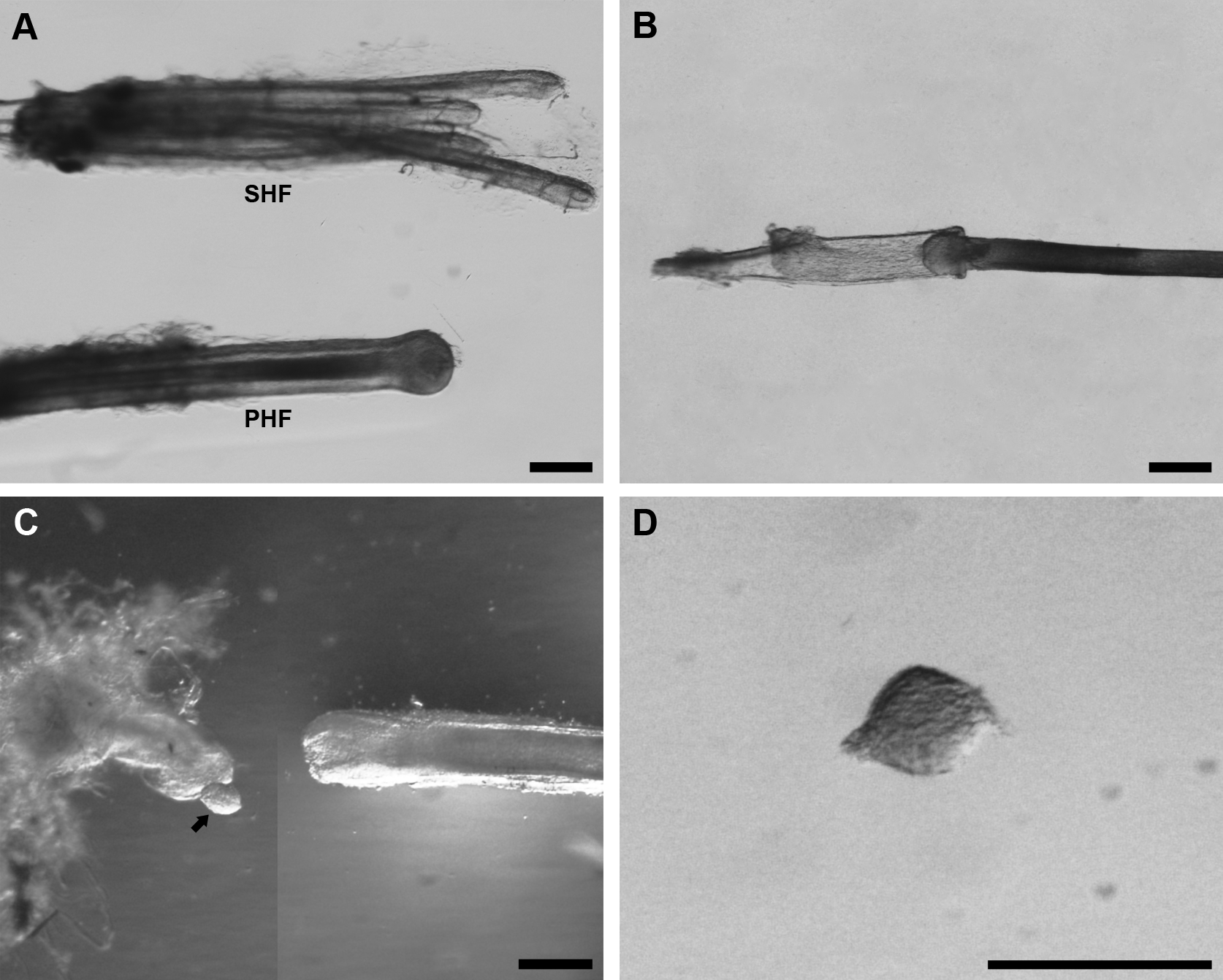

Supplement: Figure S1 — The process of DP microdissection. A) The originally isolated PHFs and SHFs. The SHFs grow in bunches (difficult to separate from each other) while the PHFs do not. The PHFs are spaced apart from each other by the dermal fibers in vivo. In this study, only the mature hair follicles which were easily to distinguish from each other were sampled. B), C), and D) showed the microdissection process of DP (Black arrow in C) from PHF. Scale bar = 250 µm. (TIF) [file pone.0076282.s001.tif]
